# Orthodontic bonding in special circumstances

**DOI:** 10.1038/s41415-024-7791-z

**Published:** 2024-09-13

**Authors:** Angus Burns, Annie Hughes, Michael O’Sullivan

**Affiliations:** 528940967352021927986 Consultant/Senior Lecturer in Orthodontics, Dublin Dental University Hospital, Trinity College Dublin, Ireland; 046738111500504247160 Postgraduate in Restorative Dentistry, Dublin Dental University Hospital, Trinity College Dublin, Ireland; 827057803814214186968 Consultant/Professor in Restorative Dentistry, Dublin Dental University Hospital, Trinity College Dublin, Ireland

## Abstract

This clinical paper provides an in-depth exploration of advanced techniques for bonding orthodontic attachments under special circumstances. Challenges arise when bonding brackets to non-enamel surfaces, such as dental restorations, and in conditions such as amelogenesis imperfecta, which affect enamel integrity. Distinct approaches required for bonding to different restorative materials, including glassy ceramics, zirconia, resin composites and metals, are outlined. Moreover, we describe strategies to manage bonding in conditions including amelogenesis imperfecta, hypodontia and microdontia in a multidisciplinary context.

## Key points

- Awareness of the precise composition of existing dental restorations can be invaluable as it will inform the approach to bonding orthodontic attachments. This is especially the case with ceramic and resin composite restorations.
- Conditions affecting the dental structure, such as fluorosis, amelogenesis imperfecta and dentinogenesis imperfecta, have highly variable presentations. The required approaches to attaching orthodontic components to these teeth range from normal enamel bonding protocols to specialised modifications.
- Patients with existing dental restorations or enamel conditions need to be made aware of both the increased risk of attachment bond failure and risk of damage to the restorations and/or teeth as a consequence of fixed appliance orthodontic treatment.

## Introduction

Successful use of orthodontic fixed appliances relies on a secure and predictable attachment of the fixed components to the teeth. Bonding of orthodontic brackets to teeth has superseded banding since the acid-etch technique was adapted for direct bonding of brackets to enamel.^1^^,^^2^^,^^3^ The numerous advantages of bonding include reduced plaque accumulation, patient comfort and reduced chairside time. Further reduction in chairside time and delegation of the bond-up procedure may also arise with indirect bonding procedures.^4^

Resin composite materials have proven to be the most suitable for orthodontic applications, demonstrating sufficient shear bond strength of at least 6-8 MPa, and the threshold tensile bond strength value proposed to resist functional and masticatory forces.^5^ Additionally, resin composite bonding is associated with an acceptable level of attachment failure rate of up to 5% over 18 months, while higher failure rates are associated with alternative adhesives, such as glass ionomers and resin-modified glass ionomers.^6^^,^^7^

With an increasing number of adults pursuing orthodontic treatment, there is a growing requirement to bond brackets to dental restorations, such as composite or amalgam, porcelain veneers, and crowns made of ceramic or metal alloys. Beyond these restorations, orthodontic practitioners also encounter patients who have undergone teeth whitening or with distinct dental conditions, including amelogenesis imperfecta. These conditions can result in anomalous tooth surfaces, presenting unique challenges for orthodontic treatment. We describe the various techniques, which can be employed to bond orthodontic attachments in these special circumstances.

## Bonding to regular enamel surfaces

Chemical treatment to ‘condition' enamel with acid was first described in 1955 by Buonocore, who used an 85% concentration of orthophosphoric acid (OPhA) to bond acrylic resin restorations to enamel.^1^ OPhA remains the most commonly used agent to etch enamel in dentistry, typically using at a 37% concentration for 15-30 seconds before orthodontic bonding.^8^^,^^9^ This acid etching (AE) creates microporosities and an uneven surface on the enamel by demineralising inter-prismatic enamel and prismatic enamel at different rates, which facilitates strong micromechanical bonding.^9^ Other methods to roughen the enamel surface before bonding orthodontic attachments, though not widely used, are described in the literature and include air abrasion, lasers and conditioning with maleic acid.^9^^,^^10^^,^^11^

Self-etch primers (SEPs) are bonding agents, which do not require a separate etching or conditioning step. Instead, dissolution of calcium from the hydroxyapatite crystals is followed by incorporation of the calcium in the polymerised resin by means of a methacrylated phosphoric acid ester.^12^ Various systematic reviews and meta-analyses have either concluded that there is weak evidence of a higher bracket bond failure rate with SEP compared to the conventional AE bonding technique, or that there is no clinical difference in terms of failure between the techniques.^7^^,^^13^^,^^14^

## Bonding to restorative materials

The specific challenges and associated modifications relating to each restorative material are outlined and summarised in Table 1.

**Table 1:** Summary of orthodontic bonding modifications for common restorative materials

Substrate	Pre-bonding modifications	Additional bonding agents	Examples of available products
Predominantly glassy ceramics	Sandblasting with aluminium oxide particles of <50 μm^9^	Hydrofluoric acid etching and silane coupling agent^9^^,^^16^	Ultradent Porcelain Etch and Silane Bisco Porcelain Primer Silane Coupling Agent
		Phosphoric acid and silane coupling agent^19^^,^^20^	3M Scotchbond Etchant and RelyX Ceramic Primer
		Silane coupling agent (no etching)	Reliance porcelain conditioner
Zirconia (with glassy ceramic veneer)	Polishing with pumice and rubber cup. Sandblasting with aluminium oxide particles of 50 μm^24^	Silane coupling agent^22^	Monobond-S Reliance porcelain conditioner
Zirconia (monolithic)	Roughen with diamond bur (to remove glaze)^25^	MDP-containing (10-methacryloyloxydecyl dihydrogen phosphate) zirconia primer^25^	Bisco Z-Prime Plus
Resin composite (except nano-filled)	Roughen with tungsten carbide or diamond bur^9^^,^^26^	(Standard bonding primer only)^9^^,^^26^	Transbond XT Primer (3M)
Nano-filled resin composite	Sandblasting with aluminium oxide^26^	Plastic conditioner^26^	Reliance plastic conditioner
Amalgam	Sandblasting with aluminium oxide particles of 50 μm^9^^,^^29^^,^^30^	4-META metal primer^3^	Reliance metal primer
Gold and stainless steel	Sandblasting with silicone dioxide particles of 30 μm^33^ or aluminium oxide particles of 50 μm^3^^,^^9^	4-META metal primer^3^^,^^9^	Reliance metal primer

### Ceramic restorations

Dental ceramics are widely used in restorative dentistry for their biocompatibility, natural appearance and excellent mechanical properties. Initially, dental crowns made from materials like feldspar or alumina were introduced in the early twentieth century and later, leucite was added to ceramics to reduce the thermal expansion differences between ceramics and metal alloys.^9^^,^^15^ Bonding orthodontic brackets to ceramic surfaces is more challenging than to enamel, requiring effective bonding techniques. Ceramics require specific etching methods due to their acid resistance and the success of bonding composite cement to ceramics mainly depends on the conditioning agent used.^15^ Ceramic surfaces can be treated by mechanical means, chemical, or both, using silane coupling agents to achieve chemical adhesion between the organic cement and the inorganic ceramic.

#### Chemical conditioning of ceramic surfaces

Hydrofluoric acid (HF) is a corrosive, inorganic acid, used widely in industry for its capability to etch glass, metal and silicon compounds, making it well-suited to overcome the acid resistance of dental ceramics. Etching with HF creates a microscopically porous surface that enhances micromechanical retention between ceramic and adhesive resins by selectively reacting with the glassy phases.^9^ The standard procedure involves etching the ceramic surface with 9.6% HFA for one minute, followed by rinsing with water and applying a silane coupling agent.^16^ It has also been recommended to use a neutralising agent, such as sodium hypochlorite (NaOCl), after the HF application to remove residual acidity.^9^ While HFA is effective, caution is necessary due to its corrosive nature and toxicity to living tissues. This risk relates to its toxicity, particularly to soft tissues, rather than its acidity.^9^^,^^17^ It is therefore critical to protect all soft tissues and to use a minimal amount of HF product to treat the ceramic surface and exercise care when rinsing the agent, employing high-volume suction.

**Fig. 1:**
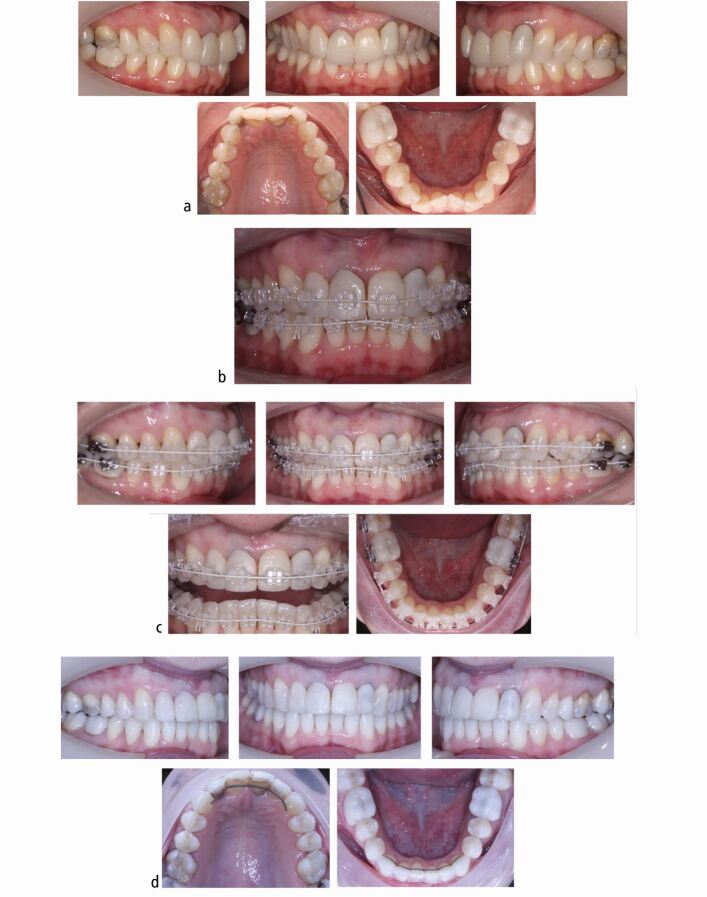
a, b, c, d) Example of a patient with all-ceramic ceramic crowns on the four maxillary incisor teeth, who is seeking correction of their dental malalignment

It has been demonstrated that using silane coupling agents, composed of hybrid inorganic-organo-functional trialkoxysilane monomers, significantly enhances the adhesion of orthodontic brackets to porcelain surfaces.^9^^,^^18^ Silane operates by establishing weak chemical connections with both organic and inorganic materials, facilitating adhesion between the porcelain and the composite resin.^3^ Following application of the silane coupling agent, a standard bonding agent and composite bracket adhesive is used (Fig. 1).

The widely accepted view that phosphoric acid is ineffective as a ceramic etching agent has also been challenged.^9^^,^^19^^,^^20^ One laboratory-based study demonstrated a shear bond strength of 6-8 MPa for orthodontic brackets bonded to naturally glazed, feldspathic, porcelain-fused-to-metal blanks, with 37% OPhA etching and silane priming.^19^ Another laboratory study comparing molar tubes bonded to porcelain molar crowns when conditioned with 9.38% HF or 37% OPhA found both techniques produced comparable shear bond strengths when a silane primer was used before bonding.^20^

#### Mechanical conditioning of ceramic surfaces

The bond strength can be further increased by mechanically roughening the porcelain surface before etching, with either a diamond bur or sandblasting with aluminium oxide particles of <50 *μ*m.^9^ Removal of the surface glaze will allow the acid to directly etch the underlying ceramic, but will also compromise the appearance of the ceramic at the debond stage.^3^ It is therefore recommended to reserve mechanical conditioning for cases where bracket bonding has failed or in joint orthodontic-restorative cases where replacement restorations are planned. Patients need to be aware of the potential compromise to the ceramic surface, which can occur when any bonding technique is used, but especially when mechanical conditioning is employed. An alternative to mechanically condition ceramic surfaces is to use CO_2_ or Nd:YAG (neodymium-doped yttrium aluminium garnet) lasers to condition the ceramic surface before silane application.^9^

### Zirconia restorations

**Fig. 2:**
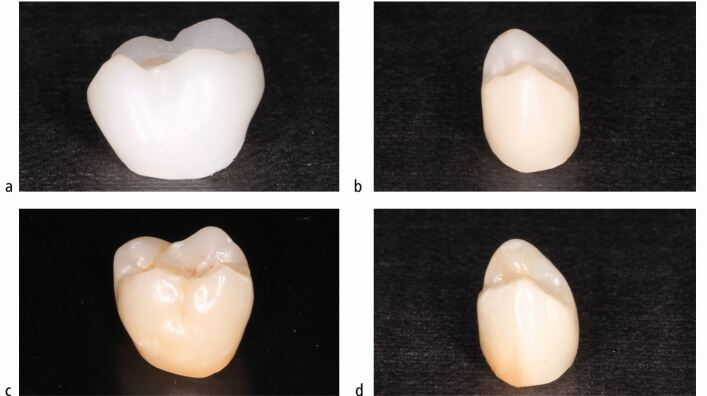
a, b, c, d) Monolithic zirconia restorations (a and b) generally appear more opaque and monochromatic in comparison to restorations with a ceramic outer layer (c and d)

Zirconia is a polycrystalline material with no glass content. It can be used clinically as a monolithic (full-contour) restoration or veneered facially with a glassy ceramic to improve aesthetics.^21^ If there is facial ceramic, the methods described above for bonding orthodontic brackets to ceramic can be applied.^22^ Full-contour restorations are commonly less aesthetic but have extremely high shear strength and are therefore generally reserved for posterior teeth. Monolithic zirconia, which will only have a surface glaze, can be clinically distinguished from a ceramic surface by its lack of translucency and monochrome appearance (Fig. 2).

Bonding to monolithic zirconia is problematic due to the absence of etchable glass and traditionally, the APC bonding concept is employed when bonding is desired. This method uses air particle abrasion (A), zirconia primer (P) and adhesive composite resin (C) steps to create adhesion.^23^

A systematic review has proposed the use of airborne particle abrasion with 50 µm Al_2_O_3_ at 0.1-0.25 MPa in combination with a phosphate monomer containing adhesive resin to condition monolithic zirconia for bonding.^24^ A zirconia primer may only offer increased bond strength for orthodontic attachment bonding when the zirconia substructure is definitely exposed and the only predictable method to remove the glaze is with a diamond bur.^25^ Dental dam isolation is also advocated during zirconia bonding.

### Resin composite restorations

Similar challenges arise when attempting to bond orthodontic attachments to surfaces, such as resin composite restorations or composite veneers. Suggested strategies to increase bond strength to existing composite include extending the etching time with OPhA to 30 − 60 seconds or mechanical roughening, either with intra-oral sandblasting or tungsten carbide or diamond burs.^9^^,^^26^ HF and silane coupling agents have been found to be ineffective and it can be expected that different composite materials will respond variably to the same conditioning technique, similar to ceramic responses.^9^^,^^27^ Simply roughening the composite surface with a bur before using a standard bonding primer has been shown to be the most effective method to achieving adequate bond strength, except in the case of nanofilled composites. In the latter, roughening by sandblasting followed by application of a plastic conditioner may be effective.^26^ Eslamian *et al.*^27^ have advocated for the use of ceramic brackets as a mitigation due to their significantly higher bond strengths. However, there is some concern that this increased bond strength could risk damaging the composite surface during bracket removal.^9^

### Amalgam restorations

Zachrisson *et al.*^28^ pioneered the practice of bonding orthodontic brackets to amalgam, recommending air abrasion with 50 μm aluminium oxide particles followed by the application of resin adhesives. Although this approach yielded a significantly lower mean tensile bond strength to that of metal brackets bonded to etched enamel, shear bond strength may be sufficient for orthodontic purposes.^9^^,^^29^^,^^30^ Attempts to enhance amalgam adhesion by surface roughening with diamond burs resulted in lower bond strengths compared to sandblasting.^9^^,^^30^ The use of a 4-META (4-methacryloxyethyl trimellitate anhydride) metal primer has been recommended to increase bond strength to existing amalgam restorations.^3^ However, the evidence to support its use for this purpose is lacking.^9^^,^^31^

Small amalgam restorations on molar buccal surfaces may not require any special treatment if sufficient enamel is available to contact the molar tube.^3^ Conversely, teeth with larger amalgam restorations are potentially more suited to banding, especially if the facility to use intra-oral sandblasting is unavailable.

### Gold and stainless-steel restorations

Using 4-META in conjunction with surface conditioning may be an effective method for bonding to various metal surfaces, such as gold and stainless steel, with sandblasting being the most effective conditioning method.^3^^,^^9^ Nonetheless, the bond strength to these metals is notably lower compared to that achieved with amalgam.^32^ Another method researched for bonding to gold, which yielded higher shear bond strength, was sandblasting the gold surface with 30 μm silicon dioxide followed by silane application. The silicon dioxide both roughens the gold surface and embeds a fine silica layer on the surface.^33^ These tests were, however, conducted in laboratory settings using standardised gold alloy.^9^ As a result, successfully bonding orthodontic appliances to these materials continues to be difficult, and banding remains a more predictable alternative.

## Whitened teeth

Tooth whitening is becoming increasingly popular as a cosmetic procedure, available both through professional in-clinic services and do-it-yourself home kits. The active constituents in dental bleaching formulae are hydrogen peroxide or carbamide peroxide. The whitening effect is achieved through an oxidative process, where peroxide compounds penetrate the tooth enamel, generating oxygen-free radicals and hydrogen peroxide ions.^34^ These agents then move through the enamel and into the dentine layer, where they act to lighten the tooth by breaking down both surface-level and deeper stains into colourless substances.^35^ After whitening, oxygen-free radicals can linger on the tooth enamel for several days. Oxygen acts as an inhibitor to the hardening process of dental composite materials. Consequently, during this period, the bonding of composite materials can be affected during the polymerisation phase.

### Adjustments to orthodontic bonding for whitened teeth

Laboratory studies suggest waiting up to three weeks after bleaching for optimal bonding but real-world studies show varied results, making it hard to pinpoint a recommended hiatus.^3^ Comparing the traditional AE technique with self-etching primers, evidence suggests that the conventional approach results in stronger bonds.^36^ One explanation is that bleaching agents may reduce the calcium and phosphate levels in enamel.^3^^,^^36^ It is, therefore, recommended to use separate etching and bonding steps, rather than self-etch priming when bonding to bleached enamel and although the evidence is equivocal, it seems prudent to advise patients not to use bleaching products for 1-2 weeks before the placement of fixed appliances.

## Dental fluorosis

Dental fluorosis results from high fluoride intake during tooth development, causing disturbances in tooth formation, with drinking water being the most common source of excessive exposure.^37^^,^^38^ Excessive fluoride during enamel formation impedes elimination of enamel proteins such as amelogenins, resulting in subsurface porosities and areas of hypomineralisation. Affected enamel can vary in appearance from white striations to brown stains or pits in severe forms.^39^ Severity can be quantified using the 10-point Thystrup and Fejerskov Index (TFI).^40^ It has been shown that resin bond strength is reduced in fluorosed teeth.^41^

### Adjustments to orthodontic bonding for teeth with fluorosis

The two mains challenges presented by severely fluorosed enamel are achieving an adequate bond strength and the risk of surface enamel fracture at debond.^41^ There is a lack of clinical evidence available to inform the best approach while the quality of the enamel varies markedly both between patients and within individual patients teeth.^3^ It is generally accepted the more fluorosed the enamel surface, the longer the etch time needs to be. Specifically, it has been demonstrated that in milder forms (TFI 1-3) enamel reacts in a similar manner to etching regular enamel with 37% OPhA, whilst more severely affected enamel (TFI 4) requires doubling the etch time (to 30-60 seconds) to produce a similar depth and pattern. For more severe forms (TFI 5+), increasing the etching time correlates poorly with the etch pattern produced and it is suggested that the superficial enamel may need to be ground away before etching the subsurface enamel to 15-30 seconds.^42^ An alternate technique is to ‘deproteinase' the surface enamel with a 5.25% NaOCl solution for 60 seconds before regular etching with 37% OPhA. This has been shown to increase shear bond strength by an average of 50% for teeth with a TFI of 4.^43^

It is important to consider that an increased bond strength may increase the risk to enamel fracture at debond.^41^ Therefore, a cautious approach would be to only employ the more aggressive techniques on a case-by-case basis and possibly only after a standard approach has failed to adequately retain brackets in patients with dental fluorosis.

## Amelogenesis imperfecta

Amelogenesis imperfecta (AI) is a hereditary condition that affects the enamel of both primary and permanent teeth. It has four principal forms:^44^

- Hypoplastic
- Hypocalcified
- Hypomature and
- Hypomaturation/hypoplastic with taurodontism.

These main forms can then be further subdivided based on mode of inheritance and clinical appearance.^44^ There is large variability in the clinical presentation from light colour change and surface irregularities to extensive enamel breakdown. Orthodontic treatment in AI presents significant challenges due to inferior bond strengths, fragile enamel, surface irregularities and lack of evidence-based clinical protocols.^45^^,^^46^^,^^47^ This can lead to multiple unwanted debonds and further breakdown of enamel during bracket removal.

The effect on enamel is different for each form of AI but abnormal crystallite formation and decreased mineral content have been reported, which may negatively affect resin bonding.^47^^,^^48^ Normal enamel is harder and has higher resin-bonding strength than hypocalcified AI enamel.^49^ Direct restorations, such as resin composite strip crowns and less-than-four-surface composite resin restorations are also more prone to failure than on unaffected teeth.^50^

### Adjustments to orthodontic bonding for teeth with amelogenesis imperfecta

Hypocalcified enamel is more porous and has lower mineral content than sound enamel.^49^^,^^50^ There is some evidence that deproteinisation with pre-treatment of 5% NaOCl may increase the bond strength to hypocalcified enamel.^51^^,^^52^^,^^53^ However, Faria-e-Silva *et al.*^47^ investigated the bond strength to extracted permanent hypocalcified AI molars and reported inferior bond strength compared to sound teeth and no improvement with the use of 5% NaOCl. Others have suggested the use of glass ionomer cement-based adhesives to improve bracket retention and prevent further enamel demineralisation.^45^ Ceramic or plastic orthodontic brackets may reduce the risk of cohesive failure of fragile enamel during debond procedures as they can be removed with a handpiece instead of pliers.^3^^,^^45^

**Fig. 3:**
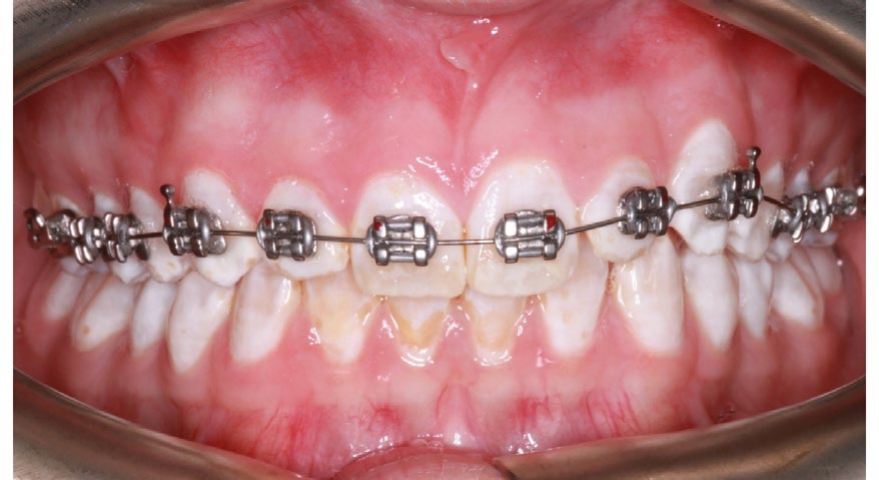
Example of a patient with mild amelogenesis imperfecta and unrestored teeth where fixed appliances are bonded directly to enamel

**Fig. 4:**
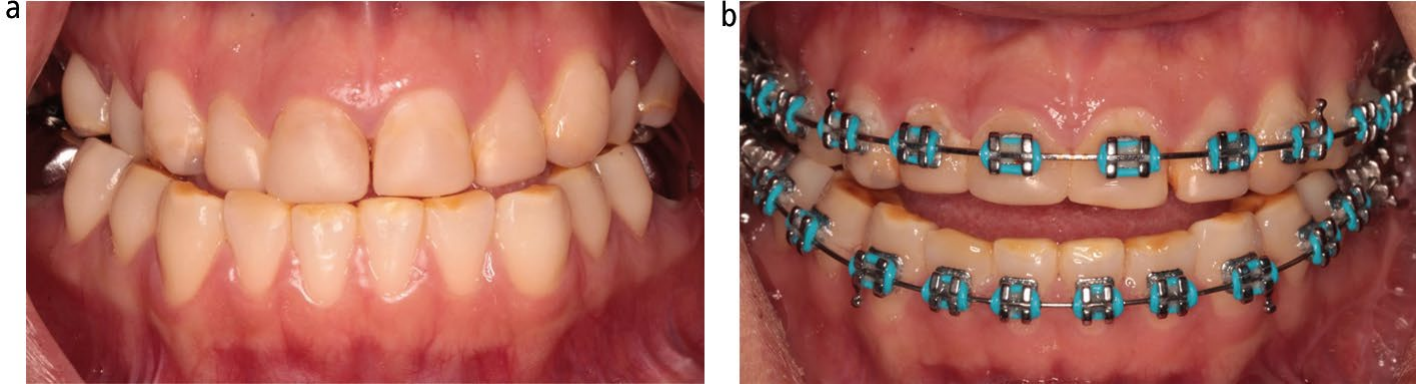
a, b) Example of a patient with hypoplastic amelogenesis imperfecta where fixed appliances are bonded directly to enamel in the mandibular arch and to provisional direct composite resin veneers in the maxillary arch. The patient is being prepared for orthognathic surgery followed by definitive indirect maxillary arch restorations

**Fig. 5:**
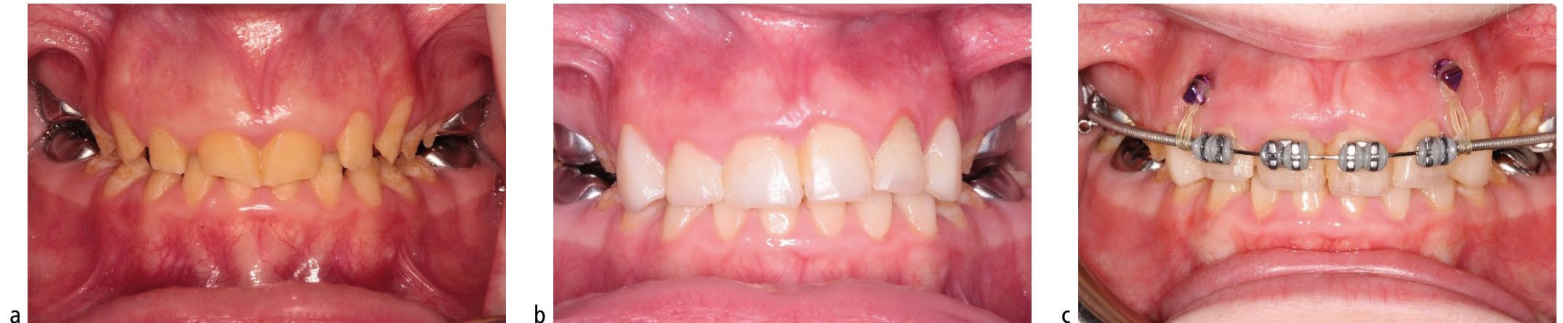
a, b, c) Example of a patient with amelogenesis imperfecta and significant loss of enamel and a deep overbite

**Fig. 6:**
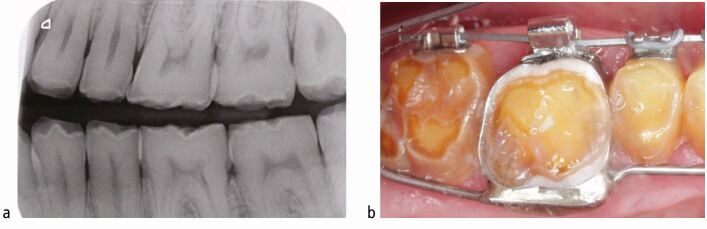
a, b) Example of a patient with amelogenesis imperfecta associated with reduced proximal enamel in the posterior teeth as seen on their bitewing radiograph. The band space will increase the available interproximal restorative space (on debond)

A predictable bond can be expected to the available enamel in hypoplastic AI (Fig. 3) as it represents a quantitative rather than a qualitative defect.^46^ It is practical, in an AI patient, to etch an easily accessible surface and examine if a clinical etch pattern is observed. However, in cases where large areas of dentine are exposed, either due to enamel breakdown or inherent quantitative defects, bonding challenges may arise. AI-affected dentine has a superficial, hypermineralised, acid-resistant layer impairing the penetration of resin adhesive, preventing resin tag formation in the tubule leading to a weaker hybrid layer.^54^^,^^55^ Epasinghe and Yiu examined the effect of additional AE on the bond strength of a self-etch adhesive to AI-affected dentine and did not find an improvement in the microtensile bond strength.^56^ Provisionally restoring such teeth with composite veneers or crowns allows for more predictable bracket bonding and also ensures better expression of the bracket prescription during the orthodontic phase of treatment (Fig. 4, Fig. 5). Similarly, in patients with a high rate of previous restoration failure, augmented retention such as banding should be considered. In circumstances where there has been loss proximal tooth structure, for example in posterior teeth affected by AI, challenges will arise when these are being prepared for full-coverage restoration due to the difficulty establishing a finish line and acceptable emergence profile for the restoration on the mesial and distal aspects for the tooth. In these cases, an addition benefit of banding the tooth (Fig. 6) is the increased interproximal distance afforded by the band space on debond.

## Dentinogenesis imperfecta

Dentinogenesis imperfecta (DI) is a rare hereditary disorder of dentine formation following an autosomal dominant pattern of transmission. It affects both formation and mineralisation of dentine in both primary teeth and permanent teeth and affected teeth appear to have altered colour and transparency - typically, amber or grey-blue, or opalescent.^57^ In mild forms, the enamel remains intact, while in more severe cases, the enamel fractures from the underlying affected dentine.

The condition is often described using the classification by Shields *et al.,*^58^ who divided DI into three subtypes:

- Type I, associated with osteogenesis imperfecta (OI) and resulting from mutations in the collagen type I genes
- Type II, presents clinically similar to Type I but in the absence of OI (though radiographically, the crowns appear bulbous with a characteristic cervical constriction and accompanied by short and slender roots)
- Type III, a phenotype characterised by large pulp chambers.

Type II is the most common variant and more recently, it has been demonstrated that Type II and Type III are essentially the same disease, associated with mutation of the dentine sialophosphoprotein gene located on the chromosome 4q21.^57^

### Adjustments to orthodontic bonding for teeth with dentinogenesis imperfecta

Research in the area of orthodontic bonding to DI teeth is limited to case reports.^59^^,^^60^ Experience suggests the enamel of intact DI teeth can be treated the same as regular enamel, though there is a risk of enamel fracturing or shearing off.^59^^,^^60^ If this is a concern, the teeth may be banded.^59^ At the other extreme, some teeth may lack sufficient structure for bonding of attachments and may require indirect restoration before orthodontic treatment.^61^ Where dentine is exposed and there is a need to bond attachments directly to dentine, a self-etching dentine bonding agent is recommended.

## Microdontia and hypodontia

**Fig. 7:**
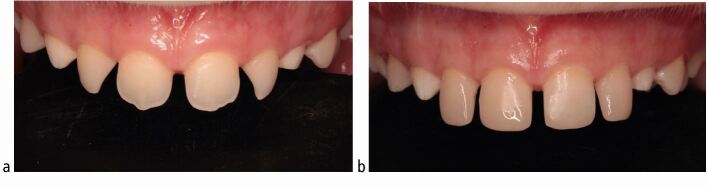
a, b) Example of a patient with microdontia affecting the upper incisor teeth. Restoring these teeth with resin composite veneers before fixed appliance treatment provides not only an immediate aesthetic improvement, but also will allow for correct expression of the bracket prescription and provide a definite end-point when closing the spaces on an arch wire

Bonding orthodontic brackets to teeth with aberrant crown morphologies exposes a limitation of pre-adjusted edgewise appliance systems. Microdontia of maxillary incisor teeth is reasonably common, with the prevalence of peg-shaped maxillary permanent lateral incisors being 1.6% in the general population, rising to 2.7% in orthodontic patients.^62^ Generally, the crowns of these teeth have a reduced size in all three dimensions. Building these teeth up to a more correct size and form with resin composite before bonding fixed appliances has several advantages apart from the immediate aesthetic improvement (Fig. 7), allowing for faithful expression of the bracket prescription and also providing a definite end-point to guide space closure.

## Conclusion

A range of special circumstances which require modification of standard techniques for bonding orthodontic attachments have been outlined. Adjuncts such as intra-oral sandblasters, along with additional conditioning etchants, bonding and coupling agents, when used appropriately, will help to increase bonding strength. Clinicians should be mindful of the variations between patients with the same developmental condition and indeed, the subtle differences between similar restorative materials in order to mitigate the increased risk of bond failure in these unique situations.
